# Sex and Text: Queering Older Men’s Sexuality in Contemporary U.S. Fiction

**DOI:** 10.1093/geroni/igaa057.3018

**Published:** 2020-12-16

**Authors:** Josep Armengol

**Affiliations:** Universidad de Castilla-La Mancha, Ciudad Real, Castilla-La Mancha, Spain

## Abstract

This paper will explore the representation of men’s aging experiences in contemporary U.S. fiction. While most gender-ed approaches to aging have focused on women, which has contributed to the cultural invisibility of older men, this study focuses on men’s aging experiences as men, thus challenging the inverse correlation between masculinity and aging. To do so, the study draws on a selected number of contemporary U.S. male-authored fictional works, which question the widely-held assumption that aging is a lesser concern for men, or that men and women’s aging experiences may be simply defined as opposed. The literary corpus includes male authors from different backgrounds so as to illustrate how (self-)representations of aging men vary according not only to gender but also class (Richard Ford), race (Ernest Gaines), and sexual orientation (Edmund White), amongst other factors. The presentation will thus end up challenging the conventional equation of men’s aging processes with (sexual) decline, exemplifying their plurality as well as irreducible contradictions.

